# Factors that Influence PRNU-Based Camera-Identification via Videos

**DOI:** 10.3390/jimaging7010008

**Published:** 2021-01-13

**Authors:** Lars de Roos, Zeno Geradts

**Affiliations:** 1 Faculty of Technology, Amsterdam University of Applied Sciences, 1097 DZ Amsterdam, The Netherlands; 2 Department of Digital and Biometric Traces, Netherlands Forensic Institute, 2467 GB The Hague, The Netherlands; geradts@uva.nl; 3 Faculty of Science, University of Amsterdam, 1098 XH Amsterdam, The Netherlands

**Keywords:** PRNU, photo response non-uniformity, source camera identification, videos, compression, snapchat, resolution

## Abstract

The Photo Response Non-Uniformity pattern (PRNU-pattern) can be used to identify the source of images or to indicate whether images have been made with the same camera. This pattern is also recognized as the “fingerprint” of a camera since it is a highly characteristic feature. However, this pattern, identically to a real fingerprint, is sensitive to many different influences, e.g., the influence of camera settings. In this study, several previously investigated factors were noted, after which three were selected for further investigation. The computation and comparison methods are evaluated under variation of the following factors: resolution, length of the video and compression. For all three studies, images were taken with a single iPhone 6. It was found that a higher resolution ensures a more reliable comparison, and that the length of a (reference) video should always be as high as possible to gain a better PRNU-pattern. It also became clear that compression (i.e., in this study the compression that Snapchat uses) has a negative effect on the correlation value. Therefore, it was found that many different factors play a part when comparing videos. Due to the large amount of controllable and non-controllable factors that influence the PRNU-pattern, it is of great importance that further research is carried out to gain clarity on the individual influences that factors exert.

## 1. Introduction

Each camera creates a highly characteristic pattern: The Photo Response Non-Uniformity pattern (PRNU-pattern). The PRNU-pattern is caused by differences in material properties and due to proximity effects during the production process of the image sensor. This pattern can be compared with various software in order to answer the following questions: ‘which camera is the source of a specific photo or video’ and ‘are certain photos or videos taken with the same camera’. After this comparison, a correlation value is linked to it, which describes the degree of similarity. In some cases, inexplicable low correlation values were measured when comparing videos. Several initiatives have already been taken by the Netherlands Forensic Institute (NFI) to determine the causes of these low correlation values. This was done by conducting small studies and proficiency tests in which international organizations participated. Since the size of these studies was limited, in most cases this matter has not been published. This study therefore made an overview of the factors already investigated. Based on this list of more than 50 different factors, three factors were chosen that could contribute to the broadening of knowledge regarding the factors that influence the PRNU-pattern. These factors include the following: compression, resolution and the length of the video. It is expected that these factors will negatively influence the PRNU-pattern, resulting in a low correlation value when a comparison is made. In previous studies [1,2,3] it was found that compression had a negative influence on the PRNU-pattern. Since Snapchats compression had not yet been investigated, this factor was chosen. Currently not much is possible in terms of getting information from the Snapchat application. A method to determine whether an image comes from the Snapchat application of a phone is therefore a welcome addition. These “Snapchat image comparisons” can also be very important to increase the burden of proof when normal reference images are missing or when large quantities of social media images have to be compared with each other. In this report the investigation regarding Snapchat serves as a starting point for further investigations. In addition to this partial study, the influence of resolution on the PRNU-pattern is being investigated. Some research has been conducted into video resolutions higher than 720p, but not enough to draw more general conclusions [3]. This partial study attempts to contribute to the formation of these more general conclusions. The last factor, the influence of the length of the video, was chosen on the basis of a recommendation that was given in a study into the influence of movement and stabilization of drones on the PRNU-pattern [4]. In this last study it was described that this factor may contribute to the deterioration of the PRNU-pattern. This paper therefore looks at three different factors that can influence the PRNU-pattern and with that the correlation value that comes from the comparison of these PRNU-patterns. The factors that have already been investigated by the NFI are also included. The aim of this research is therefore to determine which factors may provide low correlation values when comparing videos. It evaluates the computation and comparison methods used, under variation of these certain factors.

Now that an introduction has been given, the rest of this paper consists of the following: The chapter state of the art describes the basics of PRNU-investigation. The materials and methods chapter gives information regarding the choices made. The results are presented and later discussed in the chapters results and discussion. Subsequently, a conclusion has been formulated. All chapters are written by Lars de Roos, under the supervision of Zeno Geradts.

## 2. State of the Art

### 2.1. Photo Response Non-Uniformity

Photo Response Non-Uniformity is a way in which errors in the output of the image sensor are expressed [5,6]. PRNU describes the difference between the actual response of the image sensor and a uniform response [7]. During the production process PRNU occurs due to the impurity of the raw material or by the variation in size of the photodiode due to proximity effects. Since PRNU is caused by these physical properties, the characteristic differences cannot be eliminated [7]. Furthermore, the amount of noise depends on the light: if there is a lot of light, or if settings are used that let much light enter the camera, this will lead to a lot of noise. The differences and variations that arise create a noise pattern (also called a PRNU-pattern). This pattern is present in every photograph that the image sensor produces. The pattern is often seen as the “fingerprint” of the image sensor, and therefore also of a camera [8,9]. The production of the fingerprint of the camera has grown over the years to be the golden standard when comparing digital images. 

The PRNU-pattern can be made visible with advanced software, such as PRNUCompare [10]. With this software the source of an image can be retrieved. This is done with the same steps as described by Meij and Geradts [2]. In this study, steps 4 and 5 whereby the zero mean and Wiener filter are used to remove noise and artifacts created due to compression, were skipped in order to investigate the influence of compression. Reference cameras are needed to make reference images, also called flatfield images. These are images of a gray surface where the light is distributed as evenly as possible over the pixels of the image sensor. The PRNU-patterns that come from the reference images can then be compared to the images whose source has to be retrieved. In the software, such a comparison can be performed. A correlation value is calculated for this comparison which describes the degree of similarity between the PRNU-patterns.

### 2.2. Related Work

Multiple studies have been conducted over the past few years regarding the analysis of camera images. In the early stages these were mainly focused on the possibilities that Fixed Pattern Noise (FPN)—which includes Photo Response Non-Uniformities—had to offer [5,6]. Furthermore, it was also discovered that it was possible to identify a camera on the basis of pixel defects [11]. Ultimately, the method of Photo Response Non-Uniformities was further developed. For example, more complex filters and algorithms were introduced [12,13,14]. Due to further developments within this subject, even images of poorer quality could be analyzed. [15,16,17]. The goals were also adjusted. In addition to identifying the camera, it became possible to identify fake images [18,19,20,21]. Even before the turn of the century it was possible to identify a video camera on the basis of videos. However, this did not concern current digital videos but video tapes [22]. The identification of current digital videos started around 2007, when it was found possible to identify a camcorder using PRNU [23]. From that moment on, developments have progressed, and it became possible to prevent the copying and illegal downloading of movies [24]. The emergence of drones, smartphones and social media has led to yet another change in the playing field of digital images. To keep up with this, several studies have been conducted in recent years concerning smartphones, WhatsApp, YouTube and drones [2,4,25,26]. For example, it is now possible to identify the brand and model of a smartphone via video analysis [27]. In most literature that has been discussed so far, there is no explicit mention in the results, or in the interpretation and discussion of those results, that there were problems with, for instance, factors influencing the (PRNU-)patterns. Unfortunately, despite all the rapid advances, these problems can still occur. These problems are also referred to as (unexpected) artifacts or defects [28,29]. Observations made in the “Dresden Image Database” study revealed several of those artifacts [30]. In many other studies the defects are seen as beneficial since this increases the characteristic value of the noise pattern [29]. In order to identify more factors that influence the PRNU-pattern, a large number of studies have been done by the NFI. To provide insight into this, a table is made in which all the factors, and their influences on the PRNU-pattern, have been presented. In Table A1 the distinction is made between six different groups: type of camera, resolution, compression, digital processing, physical adaptation and other factors. Examples of previously investigated factors are the influence of the framerate [31], the influence of compression and resolution of YouTube videos [3,32,33] and the influence of stabilization and movement of drones [4].

## 3. Materials and Methods

The most important information about the PRNU-pattern, including a brief overview of studies that have been conducted in recent years into (factors influencing) the PRNU-pattern, has just been discussed. This knowledge is applied in this chapter to determine the research method. In this way an attempt has been made to exclude most unwanted influences and to create the opportunity to examine only the chosen factors. This chapter successively describes the camera, software and images used.

### 3.1. Camera

An iPhone 6 was used for this study. This iPhone was chosen because it had the ability to adjust the resolution, so videos could be made in 720p and 1080p, both with 30 fps (30 frames per second). No updates were made at the time of the investigation. The Snapchat application was also downloaded on this iPhone.

### 3.2. PRNUCompare

Software program “PRNUCompare” was developed by the Netherlands Forensic Institute (NFI) in order to answer the following questions: 1: Which camera is the source of a specific photo or video? And 2: Are photos or videos taken with the same camera? PRNUCompare can analyze individual or multiple photos and/or videos, including YouTube clips. It is equipped with a large selection of advanced algorithms which, when they are combined, have the ability to analyze multiple images simultaneously. Different filters can be chosen to obtain the PRNU-pattern: 2nd order extraction filter (FSTV), 4th order extraction filter and wavelet denoising/filter [2,10]. The 2nd order extraction filter (FSTV) works best for videos relative to the other filters [2,3]. The differences between the filters are mainly based on the relationship between speed and quality. In PRNUCompare it is also possible to use “frame averaging”. During the examination of all factors, the 2nd order extraction filter was used and 1 in 10 frames was extracted each time (frame averaging). When interpreting the results, the NFI uses a minimum correlation value in order to make a reliable statement about finding the source of an image. This correlation value, which can be between 0 and 1, has to be at least 0.15. In this study, this value was also used to draw conclusions about the reliability of the correlation values of the factors studied. The correlation value is a result of the equation below, which is carried out using PRNUCompare. NCC stands for the normalized cross-correlation. This is a matrix of the values between *X* and *Y* (in short, the coordinates of an image) [34]. In the rest of the article, PRNUCompare is referred to as ‘algorithm’.

(1) $$NCC\left[i,\;j\right]=\frac{\sum_{k=1}^{m}\sum_{l=1}^{n}\left(X\left[k,l\right]-\bar{X}\right)\left(Y\left[k+i,l+j\right]-\bar{Y}\right)}{∥X-\bar{X}∥Y-\bar{Y}∥}$$

### 3.3. Images

Different amounts of videos were used for the examined factors, an overview of the images per factor examined can be found in Table 1. This table also explains the type of videos (flatfield or natural) that have been used. All videos were made with the rear camera of the iPhone 6, without filters and other custom settings. All videos taken with the iPhone were stationary flatfield images, which means that the videos all consisted of a still shot of a grey background. This also insured that the light distribution was as favorable as possible and that influence on the pixels was minimal. The standard video format for Apple devices (.mov) was used, which may not be representative of non-iOS devices such as Samsung or Huawei. The videos used for the investigation of the compression of Snapchat and the resolution were all between 10 and 11 s long. Before the start of the investigation into the compression of Snapchat, it was first investigated whether there was actually a compression. This was done with images made with the Snapchat application on the iPhone 6. There were two sets, or rather parts, of Snapchat videos made: part 1 consisted of 7 flatfield videos and part 2 consisted of 15 flatfield videos. The two sets of videos were made on two different days. 

When researching the length of the videos, the first set consisted of 10 videos with different lengths (10, 14, 15 and 16 s) and the second set consisted of 10 videos with a length of 10 s. QuickTime Player was used to shorten the videos with different lengths to a length of 10 s. 

During the production of all videos, it was taken into account that the factors, from Table A1, might still have an influence on the results of the examined factors. For instance, camera settings and the amount of light. To limit these random and systematic errors as much as possible work was carried out in the same lab, in this lab use was made of controlled light, air and temperature conditions. The same device (the iPhone 6) was used and this device was in the lab at all times. Camera settings have remained unchanged, except for the change in resolution. The settings were adjusted, through the settings of the iPhone 6, from the standard 1080p with 30 fps to 720p with 30 fps and later back to 1080p with 30 fps for the other examined factors.

### 3.4. Snapchat: Extraction and Comparison of Snapchat Images

Snapchat, together with Instagram, Facebook and Twitter, is one of the most used social media in the world. In Snapchat it is possible to take photos and videos, with or without the large amount of filters and augmented reality (AR) techniques that Snapchat offers. 

Both the images and the videos were created in the same way in Snapchat and stored on the iPhone 6. Since Snapchat offers no option to save photos directly on a smartphone, the following method was chosen: first the image or the video was made, it was saved in “memories” and then exported to the photo application of the iPhone 6. The images were then taken from the iPhone to investigate on a desktop which had the PRNUCompare software. Here the images were compared to each other and to images and videos from the iPhone 6.

### 3.5. From Images to Results

It is important to zoom in a little further on what happens between the production of the images and obtaining the results of the comparisons in the form correlation values. After the images are structured in a way that is easy to load into the algorithm, the images are converted as batches to PRNU-patterns. As discussed above, certain settings and factors are taken into account and the method of Meij and Geradts is used [2]. After the images have been converted to PRNU-patterns, it is possible to perform comparisons. The patterns are compared one by one on the basis of similarities between the noise pattern, using the aforementioned equation [34]. It is possible to compare a single pattern with a single other pattern, but it is also possible to perform an entire set of comparisons directly. In the latter case, a certain number of other patterns are compared per single pattern (for example 1 vs. 20). This creates a kind of “ranking” of the best matches per pattern based on the correlation value. As mentioned, a correlation value is generated for each performed comparison, which is displayed in a table and in a graph. These tables can be exported in Excel, after which a visual presentation can easily be made, as can be found in the results of this paper.

## 4. Results

For interpreting the figures that can be found in the results this information may be relevant: the highest and lowest correlation values are indicated in each figure, these are the values of mutual comparisons of images from the same telephone. The negative (red) result therefore relates to the lowest correlation value that came from a mutual comparison between two images of the same telephone. This result is considered negative since it would be “normal” if there was no or very little difference between mutual comparison of images from the same phone (with the same settings). 

### 4.1. Resolution

**Figure 1:**
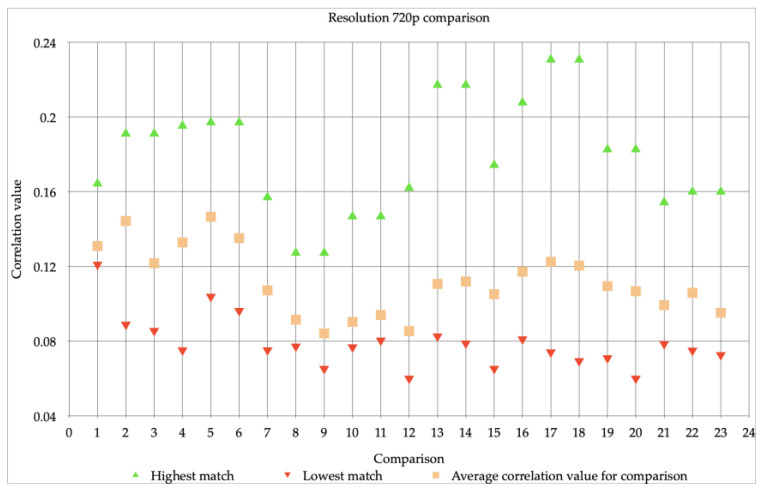
23 comparisons, done with images made with an iPhone 6, all with a resolution of 720p.

**Figure 2:**
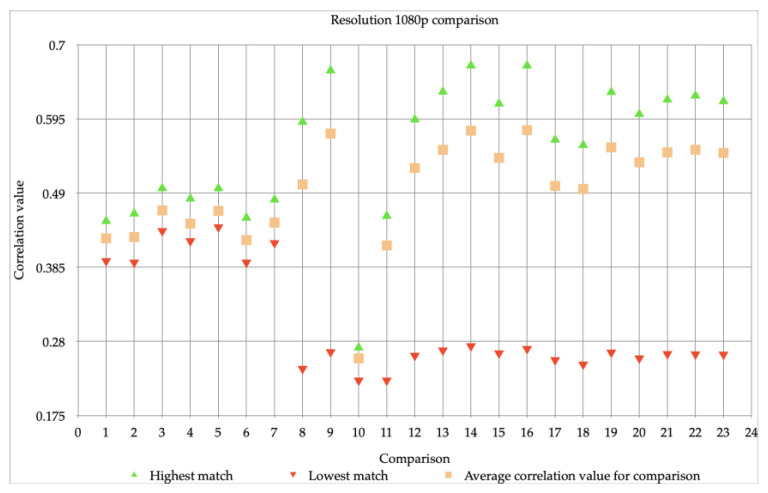
23 comparisons, done with images made with an iPhone 6, all with a resolution of 1080p.

First, we investigated whether the resolution of the videos could have an influence on the correlation value, and therefore would influence the comparison of visual material. Since previous studies only looked at a maximum resolution of 720p, we chose devices that had the ability to make videos with resolutions higher than 720p. Therefore, we decided to use an iPhone 6 video with resolutions of 720p and 1080p, both with 30 fps. Table 2 and Figure 1 and Figure 2 show the correlation values of the different video comparisons made using the algorithm. One video comparison means that different videos with a same resolution from the same device, the iPhone 6, are compared (with a one-to-one video comparison). This resulted in a highest, lowest, and average correlation value per comparison. Since the algorithm always takes the same picture into the equation, a maximum correlation value of 1.00 is always achieved. This value was omitted here as it had to be investigated how well the other images of the same device could be matched. 

In Figure 1 all 23 comparisons have a comparable lowest correlation value and the average correlation value varies a little. The highest correlation values per comparison vary more. In Figure 1 comparison 1 has a little spread, compared to the other comparisons. In Figure 2 the first seven comparisons are very close together, the rest of the 23 comparisons are more scattered. The highest and lowest value are much further apart. Comparison 10 is noticeable; it only has a highest correlation value of 0.27. 

When looking at the average correlation values in Figure 1 and Figure 2. It shows that these average correlation values of the resolutions are very different. The images with a resolution of 1080p have a much higher correlation value for all comparisons than the images with a resolution of 720p. Furthermore, the videos with a resolution of 720p cannot be reliably matched several times, they do not meet the requirement of a minimum correlation value of 0.15 used by the NFI. The difference with the videos with a resolution of 1080p is big, since a valid match can always be made with this resolution. The stability of the correlation values also differs. For example, the highest and lowest correlation values of the images with a resolution of 720p fluctuate more, this may have to do with the influence that stabilization and/or movements had during the making of the videos. Videos with a lower resolution seem to be more sensitive to this, as a result of which the correlation values differ. At higher resolutions, in this case at a resolution of 1080p, this mutual difference is much less (almost minimal). What can be concluded of this is the following: as the resolution improves, it becomes increasingly possible to obtain a PRNU-pattern (that is more resistant to influence by other factors) from a video. This makes it possible to carry out a reliable comparison with the algorithm.

### 4.2. Snapchat Compression

Subsequently, research was done into the compression of Snapchat. In order to determine whether Snapchat made any adjustments at all, a small investigation was conducted into the differences between normal iPhone 6 images and Snapchat images (which were also made with an iPhone 6). It turned out that when Snapchat was used, the resolution was adjusted to 720 × 1280. This could be caused by a different utilization of the image sensor within the iPhone 6. The resolution of the normal iPhone 6 images was 3264 × 2448 (pixel height: 3264 and pixel width: 2448). For this reason, no direct comparison could be made.

The Snapchat images could be compared to each other, but it was noticeable that the correlation values were all far below the limit of a possible match. This made it clear that Snapchat makes very big adjustments to images. The algorithm could not recognize that the images were all made by the same phone with the same Snapchat application. After that it was investigated whether Snapchat makes a compression on videos.

**Figure 3:**
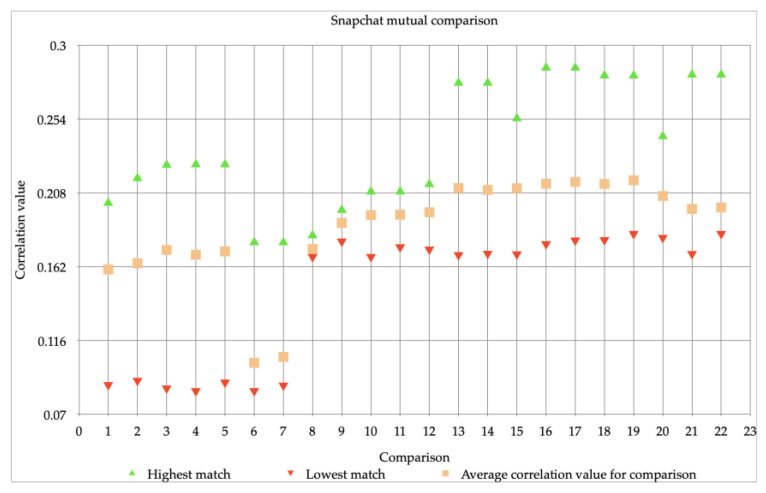
22 comparisons, done with Snapchat images made with an iPhone 6, all with a resolution of 720 × 1280.

In Figure 3 the 22 video comparisons of the Snapchat videos are shown. In this case, one single video comparison means that several videos of Snapchat from the same device, the iPhone 6, are compared. The highest correlation value is displayed with green; this value varies between 0.17 and 0.28. Orange shows the average correlation value for the comparison performed this value also varies. Correlation values between 0.10 and 0.21 have been measured here. Red indicates the lowest correlation value; these values vary between 0.08 and 0.18. In Figure 3 it is striking that there is a difference between the first 7 comparisons (part 1) and the last 15 comparisons (part 2). The lowest correlation value here is much lower than that of comparisons 8 to 22. Additionally, in comparison 6 and 7 the highest correlation value is lower than in the other 20 comparisons. There is no direct explanation for these results. Because very many factors have been taken into account, the conditions have been kept as equal as possible, see the materials and methods section. Yet it seems that making images on two different days can still cause a slight difference, even if the circumstances have remained the same.

To determine whether a match could be found on the basis of regular images of the iPhone 6, the videos that were used in researching the resolution in this study were used to perform various comparisons. Thus, the videos of the resolution study all served as reference images. 

**Figure 4:**
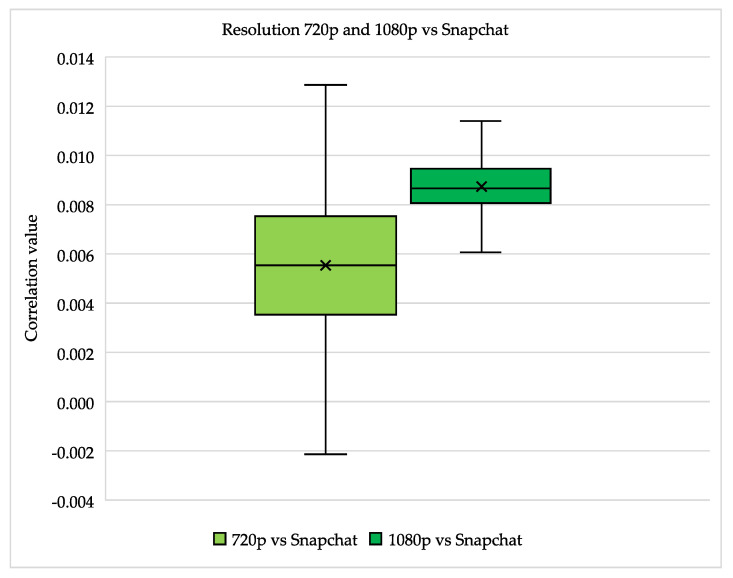
Boxplot of two times 506 individual comparisons between Snapchat images, all with a resolution of 720 × 1280, made with an iPhone 6 and images, all with a resolution of either 720p or 1080p, made with an iPhone 6.

The previous figures (see Figure 1, Figure 2 and Figure 3) showed the individual comparisons, with the highest, lowest and average values. The results that can be found in these figures can be seen as check whether the images can be matched (and thus meet the requirements set in the method). Because the correlation values, with some exceptions, were sufficient to perform mutual comparisons, the images were then compared with each other: the iPhone 6 videos with a resolution of 720p, as well as videos with a resolution of 1080p were compared to Snapchat videos. The result of these comparisons can be found in Figure 4. 

The spread of the comparison between images with a resolution of 720p and Snapchat is larger than that of images with a resolution of 1080p and Snapchat. This is not comparable to the difference already seen between Figure 1 and Figure 2, in which it became clear that images with a resolution of 1080p achieve higher correlation values, but also fluctuate on a larger scale, namely between 0.22 and 0.67 (see Figure 2). In Figure 4 this spread, when comparing 1080p videos to Snapchat videos, is between 0.006 and 0.012. Which immediately shows the decrease in the correlation values and thus the decrease in the reliability of the comparisons performed. The same drop can be observed when comparing the 720p videos to Snapchat videos, the correlation value there is between −0.002 and 0.013 instead of 0.06 and 0.23 (see Figure 1). In all cases, the highest correlation value is below the limit used by the NFI when it comes to a reliable comparison with the algorithm. 

With regard to the comparison between 1080p videos and Snapchat, it is striking that not only the spread is smaller, but also the average is higher. The 1080p vs. Snapchat comparison average is around 0.008, while it is 0.005 for the 720p vs. Snapchat comparison. This was unexpected since it was expected that this comparison would not be possible due to the difference in resolution between the Snapchat videos (720p) and regular iPhone 6 videos with a resolution of 1080p. However, slightly higher correlation values were measured in the 720p vs. Snapchat comparison. Apart from the outlier, all values in the 1080p vs. Snapchat comparison are below 0.012. In Table 3 the above mentioned highest, lowest and average correlation values of both comparisons are shown. Here it becomes clear again that no reliable comparison could be made. None of the values came close to the limit of 0.15 used by the NFI.

**Figure 5:**
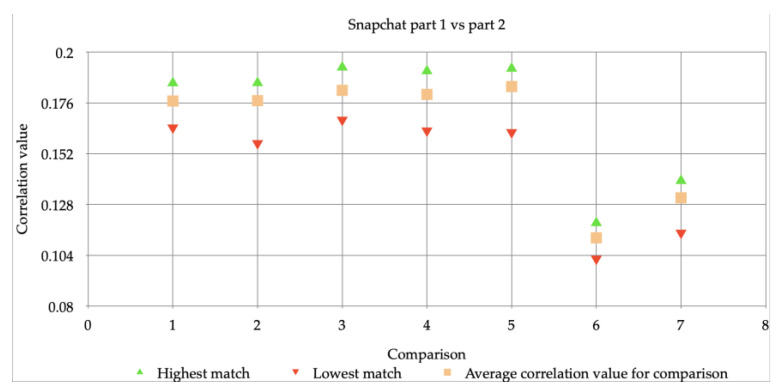
Mutual comparisons of Snapchat video’s (part 1) and Snapchat video’s (part 2).

Thus, it was found that Snapchat was making a major adjustment, not only on photos, but also on videos. It also became clear that, partly due to this adaptation, the comparisons with regular iPhone 6 videos of both 720p and 1080p could not contribute to the reliable matching of the Snapchat videos. For this reason, it was investigated in which way it could be determined whether the camera of the iPhone 6 had made the Snapchat images. A comparison has been made between both sets of the Snapchat videos: part 1 vs. part 2. In Figure 5 this comparison between the first and second set is shown. The following results were found: The highest correlation value (correlation value) varies between 0.12 and 0.19. The average correlation values, between 0.11 and 0.18, for the comparisons are close to the highest values. The lowest correlation value varies between 0.10 and 0.17.

Aside from comparisons 6 and 7, this means that the comparison between Snapchat exceeds 0.15 and can therefore be seen as “reliable”. The difference in the mean correlation value with the comparisons with the two resolutions (720p and 1080p) is at least 0.17.

### 4.3. Length of the Video

The last factor consisted of the influence of the length of a video. As mentioned earlier, only flatfield images were used here. The results of the comparisons made can be observed in Table 4. Here it becomes clear that with shorter videos (with a length of 10 s) a lower correlation value arises. The correlation values of the videos with a length of 14, 15 and 16 s are relatively close to each other. These results correspond to the following expectation: if a video is longer, it contains more frames (single images). So, if the video is longer and the algorithm extracts a pattern every 10 frames (three per second in this study, since the framerate is 30 fps), more patterns can be extracted from one video. This creates a more reliable PRNU-pattern since the average pattern over all frames of that single video is more stable. 

To investigate whether the length of the videos caused a direct difference in the correlation values during the comparison, the same videos of the iPhone 6 were investigated. These videos were cut to a length of 10 s with QuickTime Player. Table 4 shows the results of the mutual comparison of the cut videos. Here you can see the correction values of the comparison of the iPhone images when they were cut to a length of 10 s. This table clearly shows the difference between the videos that are cut and those that are not. The highest correlation values of the videos of 10 s are almost all about 0.05 (or more) lower than the videos of different lengths. The same applies to the lowest correlation values for the cut videos, which are even >0.10 lower than the videos with different lengths. What is also striking is that the measurements of the videos of 10 s are all very close to each other compared to the videos with different lengths. This is possible because a longer video provides more information about the PRNU-pattern, so that more differences can be detected. Even the video that was already 10 s long was cut to exactly 10 s. As a result, a small difference has arisen. In the Table 4, for both the comparison of the images with a different length and the images with a length of 10 s, the maximum correlation value that the algorithm always calculates is omitted. This was done because this value was unimportant in this study, as it was investigated how well the images of the same device could be matched.

Finally, it was investigated whether a cut video could be matched with the accompanying full video. This has not been processed in a report in this way before and is therefore interesting. The results of this study are shown in Table 5. The comparison performed is the following: videos of different lengths (1a to 10a) vs. videos of 10 s (1b to 10b). Table 5 shows whether the match was successful and which correlation values could be linked to these comparisons. In these results it can be seen that in some cases even a 0.99 correlation value has been achieved, which is exceptionally high. However, this has the following reason: these two videos (video 6 and 7) were already 10 s, only a few milliseconds were cut out, so only a very small adjustment was made. This has led to the exceptionally high correlation value of almost 1.00. The other correlation values also offer a positive result: it is more than clear that the algorithm is able to match cut videos to the original images.

## 5. Discussion

In the investigation into the differences between resolutions and their influence on the PRNU-pattern, two comparisons stand out: Comparison 1 of the video comparison with resolution 720p has a striking little spread, the smallest of all 23 comparisons. This could be due to other factors such as light (settings), which may have caused the video to be clearer than the other videos. Comparison 10 of the video comparison with resolution 1080p is noticeable, since it only has a highest correlation value of 0.27. This is lower than the correlation values of the remaining 22 comparisons. Most likely motion or light caused an unclear video, which meant that a less good PRNU-pattern could be extracted. Correlation values differ between the two resolutions, in most cases this difference is above 0.30. This is a very big difference, as this can indicate whether a match is considered reliable or not.

It was already known that lower resolutions (i.e., resolutions below 720p) resulted in reduced correlation values [3,4,32]. The same conclusion could be drawn from the experiment that was carried out. It also appeared that a lower resolution is less stable than videos of 1080p that were compared. As mentioned earlier, this may be due to the influence of stabilization and movement. In the literature nothing is known about this, but it is quite possible to imagine: at a lower resolution, fewer pixels are available to register (major) changes, so that details are missed.

It is recommended to carry out further research into even higher resolutions and to involve the framerate. As mentioned, it is possible to change the resolution settings on many smartphones, it is also possible to adjust the framerate: this increases the number of frames per second, which may result in an even more stable PRNU-pattern. Further research on multiple devices might help to increase the reliability of this experiment.

In the experiment on the influence of the compression performed by Snapchat, it quickly became apparent that a major adjustment was being made. This adjustment was visible on both images and videos that were created with the Snapchat application. This was not inconceivable as already known from previous studies that social media applications almost always make adjustments to visual material [1,2,3,25]. It was striking that Snapchat lowers the resolution to 720p with videos. The comparisons with the videos of 720p gave a slightly higher correlation value than the comparisons with the videos of 1080p. This can be explained by the fact that the resolution of the Snapchat videos (standard 720p) corresponds to the videos with a resolution of 720p, so that a more equal performance can be seen. What emerges from these two comparisons is that the algorithm cannot make a reliable comparison between videos that were made with Snapchat, and those that were not. So, if there is a Snapchat video that needs to be investigated whether it comes from a certain device, it is recommended not to include reference pictures of the device itself in the comparison. However, it is recommended to create Snapchat reference images with a reference device on which the application Snapchat is installed. This allows the creation of Snapchat reference images that can be compared with the algorithm. Again, it might be interesting to investigate whether the same correlation values occur with other devices. Possibly also because there is a difference between the Snapchat that is available for iOS (the operating system of Apple) and Android (the operating system of almost all other smartphones). On Android smartphones, a “screengrab” (screenshot of what the camera receives) is made instead of making an image or video with the camera of the smartphone. Capturing with the camera is what happens on an iPhone. This leads to the higher quality of the images and videos that come from an iPhone compared to an android smartphone.

In the research into the influence of the length of a video, one video of 15 s stands out, which had a lower correlation value than the other videos of 15 s. It is unclear how this difference could have arisen in this single video. It was investigated whether there was a direct influence by shortening the length of the video and it was also checked whether the videos could still be matched to the complete videos after cutting. The videos from the iPhone 6 have been duplicated and cut to a length of 10 s, using QuickTime Player. Important here is that cutting images is actually destructive research, therefore it is not recommended to cut images to an equal length during a case study. The best solution is to make reference images of the same length as the images to be examined. Incidentally, QuickTime Player, as mentioned earlier, can still have influence on the final correlation values. Unfortunately, nothing is known about the influence of this program on the PRNU-pattern. 

Some recommendations have been drawn up on the basis of the research into the factors discussed above. It summarizes what can be taken into account or where the method could be adjusted in relation to the current state of affairs. These recommendations can be found in Table 6. 

## 6. Conclusions

From the table (Table A1) that was made, several factors were known that could be responsible for low correlation values when comparing videos. In addition to this overview, three factors were examined in this study, which lead to the following conclusions: 

Compression has a negative effect on the comparison since it leads to a decrease in the correlation value. This was already known for many programs, but not specifically for Snapchat. In this research we found that through Snapchat the images (photos and videos) can be negatively influenced, in most cases so bad that a match with a normal reference is not possible. Further research is needed to confirm whether it is actually possible that a reliable comparison can be made with reference images of Snapchat. 

The better (higher) the resolution, the better (higher) the reliability of the comparison of videos will be. This was already known with resolutions up to and including 720p. This research shows that it gets even better with resolutions of 1080p.

The longer the video is, the more reliable the PRNU-pattern that can be extracted from the video. Vice versa: the shorter the video is, the worse a PRNU-pattern can be made (however, a reliable match is still possible). It is also possible, as it turns out, to match cut videos to the original videos. Often even with a very high correlation value.

Thus, it appears from the experiments that many different factors play a part in comparing videos. Due to the large amount of controllable and non-controllable factors that influence a PRNU-pattern, it is of great importance that further research is done to gain clarity on the individual influences that factors exert. 

## Figures and Tables

**Table 1:** Overview of the amount of videos per factor examined.

Factor	Amount of Videos
Resolution Snapchat Video length	23 videos (720p) and 23 videos (1080p) 22 Snapchat videos (720 × 1080) 10 videos same length and 10 videos different length

**Table 2:** Overview of the highest, average and lowest correlation values for both resolutions.

	Figure 1 (720p)	Figure 2 (1080p)
Highest correlation value	between 0.13 and 0.23	between 0.27 and 0.67
Average correlation value	between 0.08 and 0.15	between 0.25 and 0.58
Lowest correlation value	between 0.06 and 0.12	between 0.22 and 0.44

**Table 3:** Overview of the highest, average and lowest correlation values for both resolutions compared to Snapchat.

	720p vs. Snapchat	1080p vs. Snapchat
Highest correlation value	0.013	0.012
Average correlation value	0.005	0.008
Lowest correlation value	−0.002	0.006

**Table 4:** Results of the mutual comparisons of both videos with different lengths and videos with the same length (10 s).

Name of Video	Length in Seconds	Lowest Correlation	Highest Correlation	Name of Cut Video	New Length in Seconds	Lowest Correlation	Highest Correlation
1a	15	0.669	0.711	1b	10	0.526	0.537
2a	15	0.710	0.808	2b	10	0.534	0.759
3a	15	0.709	0.819	3b	10	0.536	0.725
4a	14	0.702	0.819	4b	10	0.533	0.738
5a	14	0.703	0.814	5b	10	0.533	0.749
6a	10	0.675	0.778	6b	10	0.537	0.744
7a	10	0.669	0.773	7b	10	0.534	0.743
8a	16	0.711	0.836	8b	10	0.526	0.731
9a	16	0.710	0.836	9b	10	0.535	0.757
10a	16	0.709	0.832	10b	10	0.531	0.759

**Table 5:** The correlation values of the comparison of videos of different lengths (1a to 10a) with videos of 10 s (1b to 10b).

	1b	2b	3b	4b	5b	6b	7b	8b	9b	10b
1a	0.882	0.674	0.675	0.676	0.677	0.674	0.664	0.678	0.677	0.678
2a	0.563	0.946	0.761	0.765	0.760	0.759	0.744	0.766	0.771	0.769
3a	0.559	0.756	0.947	0.778	0.771	0.768	0.752	0.775	0.781	0.779
4a	0.554	0.751	0.767	0.960	0.772	0.769	0.751	0.777	0.782	0.779
5a	0.558	0.746	0.760	0.772	0.959	0.764	0.748	0.771	0.776	0.775
6a	0.537	0.716	0.728	0.738	0.733	0.996	0.718	0.739	0.743	0.742
7a	0.532	0.709	0.719	0.728	0.724	0.725	0.991	0.733	0.738	0.734
8a	0.561	0.759	0.773	0.785	0.779	0.778	0.763	0.948	0.797	0.795
9a	0.558	0.761	0.775	0.786	0.781	0.779	0.767	0.794	0.948	0.797
10a	0.559	0.756	0.771	0.780	0.777	0.775	0.760	0.787	0.792	0.950

**Table 6:** Overview of recommendations for implementation per factor investigated.

Factor	Recommendation
Compression (Snapchat)	With images: virtually no comparison possible due to large differences in compression. It is recommended to compare as many regular images as possible and omit Snapchat photos. With videos: comparison of Snapchat videos with regular videos of the device is not possible. It might be an option to make reference videos with the Snapchat application located on the reference device. However, further research is required to confirm this finding.
Resolution	It is recommended to compare only equal resolutions (was already known). Higher resolutions give higher correlation values but take into account the fact that comparisons of lower resolutions are still reliable.
Length of the video	When creating reference images, it is recommended to make videos of the same length as the suspicious images. The reference images may also be longer, in this way more information is extracted from the video, which improves the comparison. Videos that have been cut can still be compared, the same applies as above: it is best to use videos of the same length, or longer than the suspicious images, as a reference.

## Data Availability

Data available in a publicly accessible repository that does not issue DOIs. This data can be found here: https://drive.google.com/drive/folders/1w_vkluq1oy-lMNTXmtuTIvxSjjhqtXhH?usp=sharing.

## Appendix A

**Table A1:** Overview of possible factors that influence a decrease of the (PRNU) correlation value in videos (or images in general).

	Factor	Influence
Type of camera	Exposure	Variation large, small differences
	Focal length	Zoom = drop (no identification possible)
	Camera setting (general)	This has influence
	Aperture (with shutter speed)	Little difference
	Focus	Possible factor
	Focus (middle or angle)	Middle = standard Angle(s) = more noise, so actually better
	Framerate	Higher rate = higher correlation (when comparing same rate video to video) Different rate impossible to compare
	Frames	More = better (video/video-comparison) Video/photo-comparison not (yet) possible
	I- and P-frames	Whole video or single frames highest correlation, not I- and/or P-frames
	ISO (CCD)	ISO 100 or 200 best for comparing
	ISO (CMOS)	Everything possible, if comparison with reference is made with equal (and otherwise middle) ISO value
	ISO (foveon x3)	Variation large
	Quality (camera)	Possible factor
	Shutter speed	Variation large, shorter shutter speed = lower correlation
	Temperature (decrease/increase)	This has no influence
	White balance	Variation large
Resolution	480p	No identification possible
	720p	Depending on the camera possible or not possible
	Resolution (photo)	Low = Decrease High = Increase
	Resolution (mutual difference)	No identification possible
	Resolution (video)	Low = Decrease High = Increase
	Photos with Video (resolution)	Comparison not (yet) possible
Compression	Compression	Possible factor
	Compression (online)	Compression increase = reliability decrease
	Compression (online) 2.0	So much loss, no comparison possible
	Compression (comparison)	Using the same type of compression, otherwise the correlation value decreases
	Compression/Cropped (256 × 256)	No loss and faster, lower than this value leads to degradation
	Photos with Video (compression)	Comparison not (yet) possible
	JPEG fine vs. JPEG standard	No identification possible
Digital processing	Cropped areas	No identification possible
	Grayscale (photos)	Best way to make reference images for comparison
	Increasing image	Bad and another research says good (640 × 480 to 1920 × 1080)
	Enlarge/reduce (PC)	VGA/9M = Best way
	Enlarge/reduce (camera)	Superfine = Best way
	Reducing image	This has a positive influence on the comparison
Physical adaptation	Gimbal (drone)	In combination with lower quality camera, it leads to a decrease
	Switch camera module	This has no influence
Other factors	2nd Order filter	Best result, match will be higher
	Distance to camera	Possible factor
	Motion of image	Identification possible if reference is also in motion
	Contrasts	Bad results (Usage of homogeneous substrates recommended)
	Dark/light	Darker = Decrease Lighter = Decrease Middle = Works the best
	Halogen light	Leads to lower correlation values
	Color of reference image	Green and gray show high correlation values, red and blue show lower correlation values
	Length of video	Mutual difference = Decrease
	Light (inside/outside)	Different per case
	Light (intensity)	Possible factor
	Fluorescent light	This has no direct influence
	Aging	This has no influence
